# Direct and transgenerational effects of tetracyclines on the microbiome, transcriptome, and male mating behavior of the sheep blowfly *Lucilia cuprina*

**DOI:** 10.1093/g3journal/jkaf160

**Published:** 2025-07-15

**Authors:** Alexis L Kriete, Maxwell J Scott

**Affiliations:** Department of Entomology and Plant Pathology, Campus Box 7613, North Carolina State University, Raleigh, NC 27695, United States; Department of Entomology and Plant Pathology, Campus Box 7613, North Carolina State University, Raleigh, NC 27695, United States

**Keywords:** 16S rRNA, qPCR, tetracyclines, *Lucilia cuprina*, microbiome, fsRIDL, transcriptome, mating behavior, male fitness, flystrike, anhydrotetracycline

## Abstract

Tetracyclines are broad-spectrum antibiotics widely used in agriculture, medicine, and research. However, they are associated with harmful side effects. In arthropods, parental exposure to tetracyclines has been linked to reduced health and fitness in untreated offspring. These transgenerational effects of tetracyclines could jeopardize the success of pest control programs that use tetracyclines to control gene expression. In this study, we investigated the transgenerational effects of 2 tetracyclines, doxycycline (DOX) and anhydrotetracycline (ATC), in the blowfly *Lucilia cuprina*, a significant pest of sheep. To simulate the rearing conditions of a transgenic male-only release program, blowflies were reared on standard diet alone, or standard diet plus DOX or ATC, for 3 generations, and then reared for an additional fourth generation on standard diet alone. We used behavioral assays, 16S amplicon sequencing, and mRNA sequencing to determine how DOX and ATC influenced male sexual competitiveness, microbiome composition, and gene expression in the third and fourth generations. We found that 3 generations of DOX treatment led to lower sexual competitiveness in both third- and fourth-generation males. In addition, DOX and ATC shifted the composition of the blowfly microbiome and altered the expression of numerous mitochondria- and immunity-related genes in both generations. Our study supports an emerging body of evidence that tetracyclines exert not only direct but also transgenerational effects, and sheds light on the transcriptional and microbial responses to antibiotic exposure and removal. Our findings emphasize the need for pest control programs that use tetracyclines to evaluate the long-term effects of these antibiotics.

## Introduction

The first tetracycline antibiotic, chlortetracycline, was discovered in the 1940s (Duggar 2011), and this family of antibiotics has seen widespread use ever since, with new compounds continuing to be discovered and marketed (Heaney et al. 2019). Due to their broad-spectrum activity, low cost, and favorable safety profile, tetracyclines are used extensively in human and veterinary medicine and in agriculture (del Castillo 2013; Grossman 2016). Tetracyclines and other antibiotics are also routinely used in basic research to reduce or eliminate bacteria from study organisms (Heys et al. 2018; Kennedy et al. 2018), allowing researchers to examine the functional contributions of microbiota. For example, in arthropods, over a hundred studies have employed antibiotics to eliminate *Wolbachia* (Li et al. 2014a), revealing fundamental roles for this bacterial endosymbiont in host reproduction (de Almeida et al. 2011), nutrition (Hosokawa et al. 2010), and behavior (Markov et al. 2009).

A specialized use case for tetracyclines is the Tet-ON/Tet-OFF gene regulation system. In this system, tetracyclines are used either to inactivate (Tet-OFF) (Gossen and Bujard 1992) or to activate (Tet-ON) (Gossen et al. 1995) gene expression. Tetracyclines bind to a transcription factor, the tetracycline-controlled transactivator (tTA), which is a fusion of the DNA-binding domain from the tet repressor from *Escherichia coli* and the (HSV)-VP16 transcription activation domain. The tTA protein binds to tetracycline operator (tetO) sequences upstream of a gene of interest. In the Tet-OFF system, tTA is bound to tetO sites in the absence of tetracyclines, and transcription is enhanced. When tetracyclines are present, they bind tTA and significantly reduce affinity for tetO, thereby lowering transcription of the gene of interest (Gossen and Bujard 1992). In the Tet-ON system, tetracyclines induce conformational changes in a modified version of tTA, rtTA, that allow rtTA to bind tetO sites, activating transcription of the gene of interest (Gossen et al. 1995). Tet-ON/Tet-OFF systems allow researchers to achieve precise spatiotemporal control of gene expression. Consequently, they have been widely used for basic research in plants (Padidam 2003), animals (Lewandoski 2001; Moody et al. 2002; Driesschaert et al. 2021), fungi (Stoyan et al. 2001; Kluge et al. 2018), and bacteria (Bertram et al. 2022).

Tet-OFF sex lethal systems have also been developed to help control insect pests, including the Australian sheep blowfly, *Lucilia cuprina* (Yan and Scott 2020; Williamson et al. 2021; Arp et al. 2024), and the related New World Screwworm, *Cochliomyia hominivorax* (Concha et al. 2016, 2020). *Lucilia cuprina* is a facultative parasite of sheep (Sandeman et al. 2014), and screwworm is an obligate parasite of mammals, primarily cows and sheep, though it occasionally infests humans (Mastrangelo and Welch 2012). The larvae of both species feed on living flesh (myiasis), and infestations have a very high mortality rate if left untreated (Mastrangelo and Welch 2012). The screwworm is currently controlled using the Sterile Insect Technique (SIT), in which large numbers of insects are sterilized using radiation and released into the wild (Klassen et al. 2021). The SIT and other genetic strategies have also been considered for control of *L. cuprina* (Foster and Smith 1991; Scott 2014). When sterilized males mate with wild females, the females produce no offspring, leading to population decline. Both theoretical (Vreysen et al. 2006) and empirical (Rendón et al. 2004) studies show that the SIT is more effective if only males are released. One option for achieving male-only releases is to use a Tet-OFF system to express a gene that conditionally kills or disables females. Such Tet-OFF genetic sexing systems have been developed in multiple insect species, including *L. cuprina* and *C. hominivorax* (Concha et al. 2020; Yan and Scott 2020), as well as moths (Tan et al. 2013; Reavey et al. 2022), mosquitoes (Fu et al. 2010; Marinotti et al. 2013), and fruit flies (Heinrich and Scott 2000; Fu et al. 2007). Using this approach, transgenic insects in a mass-rearing facility are maintained on a diet containing a tetracycline-class antibiotic, ensuring the continuous production of males and females. To produce an all-male generation for an SIT release, the insects are switched to a diet without the antibiotic, ensuring that a female-killing (or female-disabling) gene is switched on in the next generation. This approach is a comparatively cheap, controllable, and scalable solution to the problem of mass-producing males for SIT. The implementation of Tet-Off sexing strains could lead to more effective control of the screwworm and sheep blowfly, along with other significant pests, such as mosquitoes, where male-only releases are absolutely required because females bite and vector disease (Klassen et al. 2021). However, the success of any pest control program using a Tet-OFF system hinges on the released males—which are the progeny of multiple generations of flies reared on antibiotics—being fit and competitive.

Exposure to tetracycline-class antibiotics has been linked to negative health and fitness outcomes in diverse eukaryotes, including plants, mammals, nematodes, and arthropods (Chatzispyrou et al. 2015; Moullan et al. 2015). For example, in arthropods, tetracycline treatment is associated with reduced male fecundity and sex ratio distortion (*Drosophila melanogaster*) (O'Shea and Singh 2015), reduced female fecundity (in the springtail *Folsomia candida*) (Graber and Fallon 2019), reduced sperm viability (in the pseudoscorpion *Cordylochernes scorpioides*) (Zeh et al. 2012), delayed development (*Drosophila suzukii*) (Yan et al. 2023), altered cuticular hydrocarbon profiles and lower attractiveness to mates (in the tsetse fly *Glossina morsitans morsitans*) (Engl et al. 2018), and elevated mortality (in the honeybee *Apis mellifera*) (Raymann et al. 2017). Similarly, doxycycline treatment is associated with developmental delays and reduced fecundity in *D. melanogaster* (Moullan et al. 2015) and the German cockroach *Blatella germanica* (Pietri et al. 2018). Although little is known about how tetracycline-class antibiotics influence the health and fitness of *L. cuprina*, treating the diet of sister species *Lucilia sericata* with other broad-spectrum antibiotics was found to radically alter larval feeding preferences, likely due to the loss of bacteria that produce attractant volatile compounds (Rhinesmith-Carranza et al. 2018).

In addition to their direct effects, several recent studies have shown that tetracyclines exert transgenerational effects; that is, the untreated offspring of antibiotic-treated individuals exhibit abnormal phenotypes. For example, in the pseudoscorpion (*C. scorpioides*), both males treated with tetracycline and their untreated F1 offspring have lower sperm viability than controls (Zeh et al. 2012). In the parasitic wasp *Asobara tabida*, the untreated F1 daughters of tetracycline-treated females possess few or no oocytes and are consequently sterile (Dedeine et al. 2001). In some cases, transgenerational effects can persist for several generations after antibiotic treatment. For instance, in the fly *Drosophila simulans*, tetracycline exposure led to impaired mitochondrial metabolism 2 generations after treatment (Ballard and Melvin 2007). Other classes of antibiotics have also been associated with transgenerational effects, including reduced body weight (in the butterfly *Pieris brassicae*) (Paniagua Voirol et al. 2020), delayed development (in *D. melanogaster*) (Fridmann-Sirkis et al. 2014), and reduced sperm viability (in the tramp ant *Cardiocondyla obscurior*) (Ün et al. 2022).

The causes of the transgenerational effects of antibiotic exposure are not well-understood. In insects, the microbiome plays a crucial role in many biological processes, including immunity, nutrition, and reproduction (Oliver and Martinez 2014; Douglas 2015), and microbes are often maternally inherited (Moran et al. 2008). Therefore, the untreated offspring of antibiotic-treated insects may inherit a disrupted microbiome, leading to phenotypic abnormalities. Few studies, however, have examined the transgenerational impacts of tetracycline-class antibiotics on the insect microbiome. In the cabbage root fly *Delia radicum* (Ourry et al. 2020) and the fruit fly *Drosophila nigrosparsa* (Weiland et al. 2022), tetracycline treatment led to shifts in microbiome composition that persisted for several generations after tetracycline was removed. Similarly, honeybees (*A. mellifera*) exposed to tetracycline had abnormal microbiomes that could be “inherited’ by naive nestmates via horizontal transmission (Kowallik et al. 2022). Nestmates receiving these dysbiotic microbiomes had altered immune gene expression and were more likely to die when exposed to chemical stressors, suggesting that tetracyclines can cause long-term or transmissible damage to the immune system by perturbing the microbiome. Tetracyclines may also exert transgenerational effects by targeting mitochondria and altering the expression of genes associated with mitochondrial metabolism. As ancient bacterial endosymbionts, mitochondria are vulnerable to several classes of antibiotics, including tetracyclines, which inhibit mitochondrial gene expression and protein synthesis (Clark-Walker and Linnane 1966; Moullan et al. 2015). In insects, direct exposure to tetracyclines has been shown to impair mitochondrial metabolism, leading to abnormal phenotypes such as damaged flight muscles (Renault et al. 2018) and delayed development (Moullan et al. 2015). Transgenerational effects on mitochondria have been observed in the fly *D. simulans*, where the F2 progeny (grandchildren) of tetracycline-treated flies exhibited lower ATP production and higher mtDNA density than controls (Ballard and Melvin 2007).

The aim of this study was to investigate the direct and transgenerational impacts of tetracyclines on the microbiome, the transcriptome, and reproduction in *L. cuprina*. We tested 2 tetracycline-class antibiotics, doxycycline (DOX) and anhydrotetracycline (ATC). These compounds are often used to control gene expression with Tet-OFF/ON systems in bacteria or mammalian cell lines (Zhu et al. 2002; Ehrt et al. 2005; Bertram and Hillen 2008; Stieger et al. 2009), and while insect Tet-OFF/ON systems typically use tetracycline, DOX and ATC may be better alternatives. DOX is more stable than tetracycline (Honnorat-Benabbou et al. 2001) and has a higher rate of cellular uptake (Katiyar and Edlind 1991); these properties permit *L. cuprina* and screwworm Tet-OFF sexing strains to be maintained at lower concentrations of DOX than tetracycline (Arp et al. 2024). ATC has a higher affinity for tTA than tetracycline (Gossen and Bujard 1992), making it a more potent effector in Tet-OFF/ON systems. It is an atypical tetracycline that apparently kills bacteria by perturbing the cell membrane rather than inhibiting translation (Oliva et al. 1992; Chopra 1994). The MIC (minimum inhibitory concentration) of ATC required to prevent the growth of *Escherichia coli* is 4 times higher than tetracycline (Chopra 1994). Indeed, ATC has been used at low concentrations (e.g. 50 ng/mL) as an inducer of gene expression in bacteria (Lutz and Bujard 1997; Ehrt et al. 2005).

In this study, we reared blowflies for 3 generations on DOX or ATC, then removed these antibiotics from the final, fourth generation. This experimental design mimics the rearing conditions found in genetic pest management programs utilizing a Tet-OFF sexing system, where the final (released) generation is not reared on tetracyclines, but preceding generations are. We used 16S amplicon sequencing and mRNA sequencing to analyze the microbiomes and transcriptomes, respectively, of third-generation (G3) flies, as well as their untreated, fourth-generation (G4) offspring. We also measured mitochondrial and bacterial DNA abundance using qPCR, and tested G3 and G4 adult males for sexual competitiveness in behavioral assays. We hypothesized that DOX and ATC exposure in G3 would reduce male sexual competitiveness and lead to less diverse microbiomes, lower levels of bacterial and mitochondrial DNA, and altered expression of genes related to immunity and mitochondrial function. We further hypothesized that the untreated G4 offspring of the antibiotic-treated flies would either resemble their G3 parents or display similar, but less extreme, phenotypes.

## Materials and methods

### Lucilia cuprina rearing

All flies (*L. cuprina*) used in this study were reared according to previously described protocols (Li et al. 2014b), with the exception of the dietary treatments applied to wild-type flies. Wild-type fly colonies were reared on 3 different diets: 25 ug/mL DOX (98% Doxycycline hyclate, Thermo Scientific), 100 ug/mL ATC (98% Anhydrotetracycline hydrochloride, Abcam), or the control diet with no antibiotic supplement (H_2_O). In addition to wild-type flies, transgenic flies carrying a red marker gene were used in this study for male competitiveness assays; these transgenic flies were reared only on the control (H_2_O) diet. The transgenic strain, EF3A (Yan and Scott 2015), was selected based on its superior performance in male sexual competitiveness trials relative to other transgenic strains tested (Supplementary File 1).

To deliver the antibiotics (DOX or ATC) to the larval diet, the compounds were dissolved in deionized water and thoroughly mixed with 93% ground beef to final concentrations of 25 ug/g (DOX) or 100 ug/g (ATC). To make up the control larval diet, an equivalent volume of pure deionized water was mixed with the ground beef (referred to as H_2_O control in the main text). All adult flies were provided with sugar and a protein-rich biscuit. The control group received water with no antibiotic supplementation; the DOX and ATC groups received water supplemented with DOX or ATC to final concentrations of 25 ug/mL or 100 ug/mL, respectively. These concentrations are representative of dosages used in TET-OFF systems developed for genetic control of the screwworm (Arp et al. 2024) and *L. cuprina* (Li et al. 2014b). DOX was used at a lower concentration than ATC in this study based on data showing that 25 ug/mL DOX was sufficient to maintain *L. cuprina* Tet-OFF strains (Arp et al. 2024), whereas concentrations of at least 100 ug/mL ATC were required to maintain a Tet-OFF strain (Supplementary Table 3).

Three independent *L. cuprina* colonies, containing N = 100 individuals per colony, were established for each of the 3 dietary treatments. The 9 colonies were reared in parallel for 3 generations on their assigned diet (DOX, ATC, or H_2_O). Each colony was then reared in parallel for an additional fourth generation on the control (H_2_O) diet. Third- and fourth-generation flies from these colonies were sampled for DNA and RNA extraction. Larvae were sampled at the third instar stage (5 d post-hatching) and adults were sampled at 7 d post-eclosion. Afterwards, additional colonies were established with the same rearing conditions (i.e. flies were reared for 3 generations on DOX, ATC, or H_2_O, and switched to H_2_O in the fourth generation), with a portion of male adults from each G3 and G4 colony set aside for use in sexual competitiveness assays. Supplementary Fig. 3 summarizes the fly rearing design for all experiments in this study.

### Male sexual competitiveness assays

Male sexual competitiveness was assayed by allowing males to mate with females and scoring female oviposition data to determine paternity. To perform the assay, 40 virgin, 7-day-old, wild-type females reared on the control (H_2_O) diet were anesthetized with CO_2_ and transferred to 13″×13″×13″ cages (BioQuip: DP1000) containing a water-soaked cotton ball. Females were given 2 h to recover in the cage. Male flies, which were similarly anesthetized and allowed to recover separately, were added to the cages containing the females at the end of the 2-h recovery period; 80 males were added to each cage, of which 40 males were 7-day-old, transgenic (EF3A) males carrying a red marker gene; these males were reared on the control diet (H_2_O). The remaining 40 males were 7-day-old wild-type G3 or G4 flies reared on DOX, ATC, or H_2_O (G3) or H_2_O (G4). Six replicate assays were performed for each group (36 assays total).

Males were given 24 h to mate with females and then removed from the cages. Females were left in the cages for an additional 24 h to promote egg maturation and then transferred individually to 50 mL plastic cups containing 10 g of 93% ground beef as an oviposition substrate. Females were given 48 h to lay eggs, which were screened for the expression of a fluorescent red marker gene. Females that laid red fluorescent eggs were scored as having mated with an EF3A male, and females that laid non-fluorescent eggs were scored as having mated with a wild-type male. To ensure that unfertilized eggs were not mistakenly scored as eggs sired by wild-type males, we monitored eggs until hatching and verified that all clutches produced viable larvae. Mixed paternity due to female remating (indicated by red fluorescent and non-fluorescent larvae hatching in the same clutch) was rare (<3% of clutches screened), and these clutches were left out of the final dataset. Male sexual competitiveness was calculated as the number of females that mated with wild-type males divided by the total number of mated females. Statistical analysis of the data was conducted in R version 4.2.2 (R Core Team 2023). A one-way ANOVA was used to detect differences in mean sexual competitiveness among the wild-type male groups, followed by pairwise t-tests to determine which groups differed from each other. *P*-Values were adjusted for multiple comparisons using the Benjamini–Hochberg correction, and adjusted *P*-values < 0.05 were considered statistically significant. The Shapiro–Wilk and Bartlett tests were used to determine whether the data met assumptions of normality and homogeneity of variance, respectively.

### DNA isolation

Whole-body larval and adult tissue samples were flash-frozen in liquid nitrogen and stored at −80 °C prior to DNA isolation. To extract DNA, samples were homogenized in 500 μL STE (10 mM Tris, 1 mM EDTA, 100 mM NaCl, pH 8.0) buffer in an HT Mini bead mill (OPS Diagnostics) for 2 min at 3200 RPM. DNA was then purified from the homogenate using a DNEasy Blood & Tissue kit (QIAGEN). Purified DNA was quantified using a QUBIT 2.0 fluorometer, and equimolar amounts of DNA were pooled between the 3 samples collected from each experimental group. The pooled DNA samples (*N* = 54 samples total) were split into aliquots for 16S amplicon sequencing and qPCR, and stored at −80 °C until use.

### qPCR

To quantify mitochondrial, bacterial, and nuclear DNA in *L. cuprina* DNA samples, we designed 3 qPCR primer pairs (Supplementary Table 4) targeting the *COXI*, *16S*, and *LcGST-1* genes, respectively. All qPCR primer pairs were tested using DNA gel electrophoresis to ensure they amplified products of the expected size. Standard curves for each primer pair were generated using a series of 6 ten-fold DNA dilutions run in triplicate, and each primer pair was confirmed to have acceptable amplification efficiency values (100–110%) and *R*^2^ values (>0.99). qPCR was performed on a Bio-Rad CFX384 Touch Real-Time PCR Detection System using Maxima SYBR Green/ROX qPCR Master Mix (2X). Each qPCR sample was run in triplicate in a 25-µL final volume containing 5 ng DNA, 1× Master Mix, and 0.4 µM each primer. The thermocycler program parameters for qPCR consisted of an initial denaturation step for 10 min at 95 °C, then 40 cycles of 95 °C for 15 s (denaturing), 57 °C for 30 s (annealing), and 72 °C for 15 s (extension). A melt curve step was included at the end of each run to verify that a single product was amplified. Cycle threshold (Ct) values were called and averaged for each triplicate sample in CFX Maestro (Bio-Rad, version 5.3.022.1030). To control for potential experimental variability in the amount of total DNA loaded into each qPCR sample, the *COXI* and *16S* gene copy numbers were normalized to the *LcGST-1* (nuclear) gene copy number. To do this, ΔCt values were calculated by subtracting the Ct values of *COXI* and *16S* from the Ct value of *LcGST-1* for each sample. ΔΔCt Values (Livak and Schmittgen 2001) were then calculated for each sample by subtracting the averaged ΔCt value of the control samples from *COXI* and *16S* ΔCt values. The control samples for each generation/stage combination were the H_2_O-reared flies belonging to the same cohort (e.g. the control group for G3 ATC- and DOX-reared larvae was the G3 H_2_O-reared larvae). Statistical analyses were performed in R using unpaired *t*-tests with Benjamini–Hochberg correction applied for multiple comparisons.

### 16S sequencing and data analysis

Library preparation, sequencing, and quality control were carried out by Novogene Corporation Inc. (Sacramento, CA, USA). The V4 region of the 16S rRNA gene was amplified using 16S V4: 515F-806R primers (Supplementary Table 4). Sequencing was performed on an Illumina NovaSeq 6000 platform (Illumina, Inc., San Diego, CA, USA). Demultiplexing and barcode removal were performed by Novogene. Approximately 100,000 paired-end, 250-bp reads were generated per sample. The raw 16S reads are available at NCBI Sequence Read Library BioProjectID: PRJNA1118283 (https://www.ncbi.nlm.nih.gov/bioproject/PRJNA1118283/).

### ASV clustering, taxonomic assignment, filtering, and normalization

Read trimming, amplicon sequence variant (ASV) clustering, and chimera removal were performed using the dada2 package (Callahan et al. 2016) in R. Reads were trimmed by removing the first 28 bases and truncating the read length to 195 bases. The dada2 clustering/denoising algorithm was then run with default parameters. Taxonomic classifications were made using the IDTAXA algorithm implemented in the DECIPHER package (Wright 2016) based on the SILVA 99% OTU v.138.1 reference database (Quast et al. 2012; Yilmaz et al. 2014). The sequences of the 50 most abundant ASVs were cross-referenced to the NCBI 16S rRNA database using the BLASTn search tool (Altschul et al. 1990) and resolved to more precise taxonomic identities when possible. Singletons (ASVs with a single read count, which are often artifacts) and ASVs that failed to be assigned to any taxonomic level were removed from the dataset, as were ASVs within the domain Archaea, order Chloroplast, or family Mitochondria. Additionally, to reduce noise in the dataset, very rare ASVs (below 0.001% total abundance, or 70 total reads summed across all samples), as well as ASVs found in only one sample, were removed. Finally, to compensate for uneven read depths across the samples, the dataset was normalized using scaling with ranked subsampling, an alternative to rarefying that preserves the original community structure, via the SRS R package (Heidrich et al. 2021). Samples were normalized to a depth of 65,135 reads per sample. The normalized dataset was used for all subsequent analyses unless otherwise specified.

### Shared and unique taxa

To visualize patterns of shared and unique taxa, we generated Euler plots using the MicEco R package (https://github.com/Russel88/MicEco; Ver. 0.9.19) with the taxon prevalence cutoff set to 0.2.

### Alpha diversity, beta diversity, and ordination

The R packages phyloseq (McMurdie and Holmes 2013), picante (Kembel et al. 2010), and microbiome (https://github.com/microbiome/microbiome; version 1.24.0) were used to calculate the Shannon Index, Faith's Phylogenetic Diversity, and Pielou's Evenness, respectively. Pairwise comparisons were performed using the Wilcoxon rank-sum test with Benjamini–Hochberg correction for multiple comparisons. The distance matrix for beta diversity analysis was computed in QIIME 2 (Bolyen et al. 2019) (q2cli version 2022.11.1) using DEICODE (Martino et al. 2019), which performs Robust Aitchison PCA to transform the data. DEICODE was run with default parameters on the non-normalized dataset, following the developers' recommendations. Statistical comparisons of beta diversity were performed using pairwise PERMANOVA with Benjamini–Hochberg correction for multiple comparisons. The DEICODE ordination data were used to generate the Principal Coordinates Analysis (PCoA) plot.

### Differential abundance testing

Analysis of Compositions of Microbiomes with Bias Correction 2 (ANCOM-BC2) was used to identify differentially abundant taxa across experimental groups with the R package ANCOMBC v.2.6.0 (Lin and Peddada 2020). Non-normalized data were used as input because ANCOM-BC2 performs normalization automatically. A pairwise directional test was run to compare experimental groups with the following parameters: prevalence cutoff = 0 (since prevalence filtering had already been performed on the dataset); structural zero detection set to TRUE in order to identify missing taxa; and the Holm correction applied for multiple comparisons. Comparisons with q-values (i.e. corrected *P*-values) < 0.05 were considered statistically significant.

### RNA isolation

Larvae and adults were randomly and individually sampled from each G3 and G4 colony for RNA isolation and sequencing. Flies were flash-frozen in liquid nitrogen and stored at −80 °C prior to RNA extraction. To extract RNA, flies were placed in 2-mL bead tubes prefilled with 1.5-mm zirconium beads and containing cold Trizol™ reagent (Thermo-Fisher). Samples were immediately homogenized in a bead mill (HT Mini: OPS Diagnostics) for 60 s at 3200 RPM. The bead-beating step was repeated 3 times for each sample, with samples being rested on ice for 20 s between each beating step. RNA was then isolated according to the QIAGEN RNeasy Mini Kit protocol for total RNA extraction from animal tissue, with the exception that we substituted Trizol™ for the kit's lysis buffer. RNA samples were quantified using a Qubit® 3.0 fluorometer (Thermo-Fisher) and stored at −80 °C until use. Three female larvae, three male larvae, three female adults, and three male adults from each G3 and G4 colony (216 individuals total) were used for downstream RNAseq analysis. Equimolar amounts of RNA from each aforementioned group of 3 flies was pooled, generating the final set of 72 samples used for RNA sequencing.

### RNA sequencing and data analysis

Illumina RNA library construction and sequencing were performed by the North Carolina State Genomic Sciences Laboratory (Raleigh, NC, USA). Samples were enriched for mRNA using oligo-dT beads provided in the NEBNExt Poly(A) mRNA Magnetic Isolation Module (New England Biolabs, USA). cDNA libraries for Illumina sequencing were constructed using the NEBNext Ultra II Directional RNA Library Prep Kit (NEB) and NEBNext Mulitplex Oligos for Illumina (NEB) using the manufacturer-specified protocol. Samples were selected for a final library size (adapters included) of 400 to 550 bp using sequential AMPure XP bead isolation (Beckman Coulter, USA). The final quantified libraries were pooled in equimolar amounts for clustering and sequencing on an Illumina NovaSeq 6000 DNA sequencer, utilizing 1 lane of an S4 150×2 PE sequencing reagent kit (Illumina, USA), generating approximately 50 M reads per sample. The software package Real-Time Analysis (RTA) was used to generate raw base call files, which were then de-multiplexed by sample into fastq files. RNAseq reads are available at NCBI Sequence Read Library BioProjectID: PRJNA1227982 (https://www.ncbi.nlm.nih.gov/bioproject/PRJNA1227982). Read trimming, adapter removal, and quality filtering were performed using fastp version 0.21.0 (Chen et al. 2018) with default parameters. Filtered reads were merged and aligned to the *Lucilia cuprina* reference genome assembly (NCBI ID: ASM2204524v1) using the STAR aligner version 2.7.9a (Dobin et al. 2013) with default parameters. The resulting read count table and metadata were imported into R for data analysis and visualization. Read count data were normalized using the variance-stabilizing transformation (vst) prior to visualization with PCA. Differential gene expression analysis was conducted in R using DESeq2 version 1.44.0 (Love et al. 2014). Non-normalized read count data was used as input for DESeq2 because DESeq2 performs normalization automatically. The false discovery rate (FDR) was set to 0.05 for all comparisons. To avoid instances in which genes with very low counts were spuriously identified as significant because they had high counts elsewhere in the dataset, a post hoc gene count filter was applied to remove any gene from an individual comparison if the majority of counts in the samples being compared were <5. Barplots and volcano plots used for data visualization were generated in R with the ggplot2 package. gProfiler (https://biit.cs.ut.ee/gprofiler/gost) was used to identify enriched GO terms among differentially expressed genes using the g:OST software tool. The g:SCS significance threshold was used as the multiple testing correction method, with corrected *P*-values < 0.05 denoting statistical significance. Gene annotation was performed using the gProfiler tool g:Convert (https://biit.cs.ut.ee/gprofiler/convert) using the Ensembl database search.

## Results

### Doxycycline reduces sexual competitiveness in treated males and untreated offspring

**Fig. 1.**
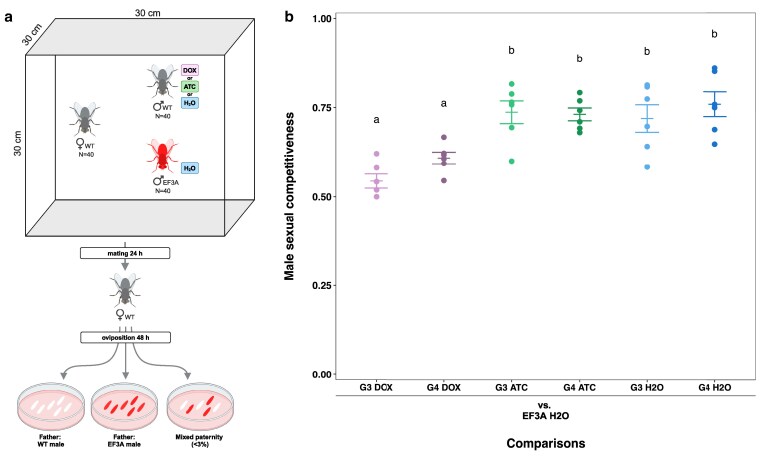
Male sexual competitiveness assays. a) Assay procedure. Virgin wild-type females were placed in cages with EF3A males (reared exclusively on standard diet [H_2_O]) and wild-type males (reared on DOX, ATC, or H_2_O for generations 1 to 3, and on H_2_O in generation 4) at a 1:1:1 ratio. After mating, females were transferred to an oviposition substrate to lay eggs, which were scored for fluorescence to determine paternity and calculate male sexual competitiveness scores. Rare instances of mixed paternity were excluded from further analysis. b) Male sexual competitiveness scores, defined as the proportion of mated females that mated with wild-type males. Six replicate assays were performed for each comparison; filled circles indicate the wild-type male sexual competitiveness score from each assay. Horizontal bars correspond to the mean +/− SEM for each comparison. Different letters above the groups indicate statistically significant differences among sample means for those groups (*P* < 0.05; pairwise *t*-test with Benjamini–Hochberg correction for multiple comparisons).

We conducted behavioral assays to determine how DOX and ATC treatment affected male sexual competitiveness in *L. cuprina*. In these assays, the ratio between treated males, untreated males, and untreated females was 1:1:1, as done previously with *C. hominivorax* (Concha et al. 2020). The untreated males carried a heritable red fluorescent protein gene, allowing us to screen the progeny of the females to determine whether they mated with the DOX- or ATC-treated males or untreated males (Fig. 1a). If the treated males were fully competitive, then about half the time, females would mate with the treated male. However, we noted that the wild-type males were inherently more sexually competitive than the transgenic competitor males used in these assays (Supplementary File 1). *Lucilia cuprina* strains were reared on standard diet (93% beef for larvae, protein cookie, and water for adults) supplemented with either DOX or ATC solution or H_2_O with no antibiotic. DOX treatment was associated with reduced competitiveness in both generations tested (G3 and G4) (Fig. 1b). G3 DOX males mated with, on average, 24% fewer females than G3 control diet males, whereas G4 DOX males mated with 20% fewer females than G4 control males. These results indicate 3 generations of exposure to 25 ug/mL DOX is sufficient to induce both direct and indirect (transgenerational) effects on male sexual performance. Conversely, ATC had no effect on sexual competitiveness in either G3 or G4 males (Fig. 1b), despite being administered at a higher dose than DOX.

### Doxycycline and anhydrotetracycline perturb the microbiome of treated flies and untreated offspring

**Fig. 2.**
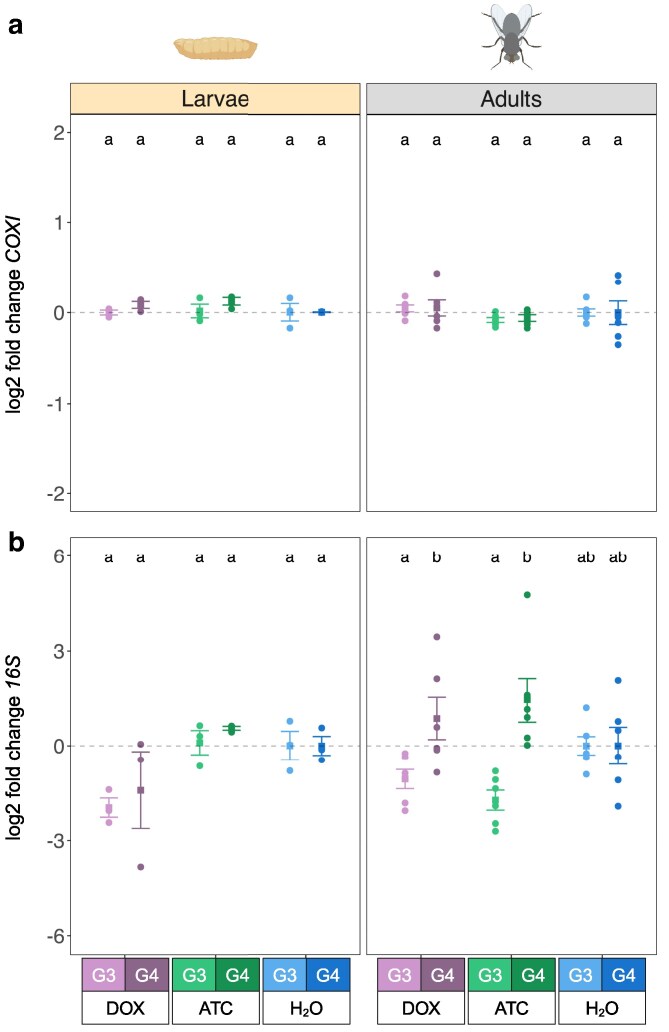
qPCR measurements of *COXI* and *16S* copy numbers in doxycycline- and anhydrotetracycline-treated flies and their offspring. a) *COXI* (mitochondrial DNA). b) *16S* (bacterial DNA). Log2 fold changes (−ΔΔCt) relative to H_2_O-reared controls are plotted along the y-axis for each gene. Individual data points (colored circles) represent the averaged log2 fold change from 3 technical qPCR replicates. Colored squares with error bars represent the mean ± SE for each comparison. Statistically significant differences between experimental groups are indicated by different letter combinations (*P* < 0.05; unpaired *t*-test with Benjamini–Hochberg correction for multiple comparisons).

We next examined the direct and transgenerational effects of DOX and ATC on mitochondria and the microbiome, given their importance to male fitness in insects. We began by using qPCR to measure the copy numbers of the *COXI* and *16S* genes as proxies for mitochondrial and bacterial DNA abundance, respectively. Unexpectedly, we found that DOX and ATC had no impact on mitochondrial DNA abundance, regardless of generation or developmental stage (Fig. 2a). In contrast, bacterial DNA abundance was influenced by diet and generation in adult flies. *16S* gene copy number was approximately 4 times higher in G4 DOX adults relative to G3 DOX adults, and 8 times higher in G4 ATC adults relative to G3 ATC adults (Fig. 2b). These results suggest that DOX and ATC reduce the bacterial load in G3 adults, but that bacteria return to, or in some cases surpass, baseline levels within one generation after DOX and ATC are removed.

**Fig. 3.**
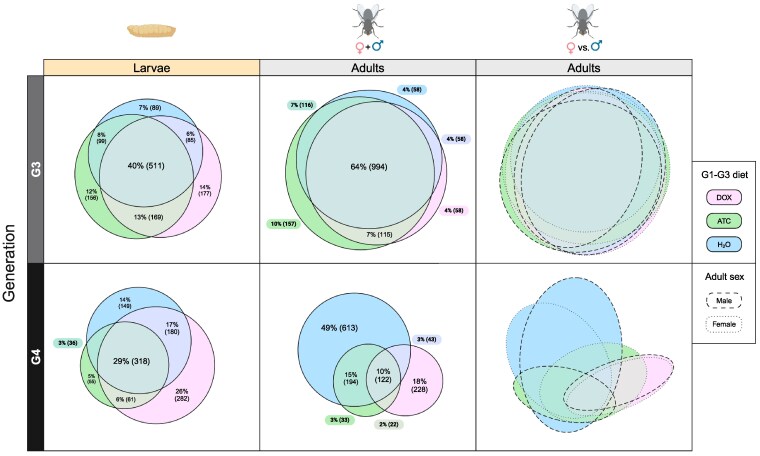
Shared and unique taxa in different experimental groups. Ellipsoids representing each group are color-coded based on the G1-G3 diet. Solid ellipsoid outlines denote mixed-sex samples; dashed and dotted outlines denote male and female samples, respectively. Percentages and numbers in parentheses indicate the proportion and number of taxa, respectively, in each group. These values are not displayed in the separate-sex adult plots (third column) due to the large number of overlapping ellipsoids.

We next asked whether DOX and ATC altered the composition of the microbiome in treated G3 flies or their untreated G4 offspring. We began by examining bacterial community overlap (shared and unique taxa) among the different experimental groups. We found that DOX and ATC altered microbiome composition in both generations (G3 and G4) and developmental stages (larvae and adults), with DOX- and ATC-treated flies and their offspring possessing dozens to hundreds of unique taxa (Fig. 3). Male and female adults tended to have similar bacterial communities, with the exception of the G4 ATC females, which were more similar to the G4 DOX adults than their male counterparts (Fig. 3). We observed a general reduction in the overall number of taxa in G4 relative to G3 samples. The G4 larvae and adults reared on standard diet (with H_2_O with no antibiotic added) had 87% and 79% as many taxa as control G3 larvae and adults, respectively. This loss of taxa from the control samples may be due to transient environmental changes in the laboratory. We observed a much steeper transgenerational loss of taxa in the G4 DOX and ATC samples, with G4 DOX and G4 ATC larvae possessing 89% and 50% as many taxa as the G3 DOX and G3 ATC larvae, respectively, and G4 DOX and G4 ATC adults possessing 34 and 27% as many taxa as their G3 parents (Fig. 3). The G4 samples also shared fewer taxa with each other than the G3 samples, despite the fact that all G4 flies were reared on the same (control) diet. Whereas 40% of all taxa were shared by DOX, ATC, and H_2_O-reared larvae in G3, only 29% of taxa were shared among these groups in G4. A more extreme reduction in shared taxa was observed in adults, in which 64% of taxa were shared in G3 and only 10% shared in G4. This divergence was due primarily to the loss of shared taxa among the G4 DOX and G4 H_2_O groups (Fig. 3). Taken together, these results show that DOX and ATC disrupt the microbial community not only in antibiotic-treated (G3) flies but also in the untreated (G4) offspring of antibiotic-treated flies, demonstrating a transgenerational effect of tetracyclines on the blowfly microbiome.

**Fig. 4.**
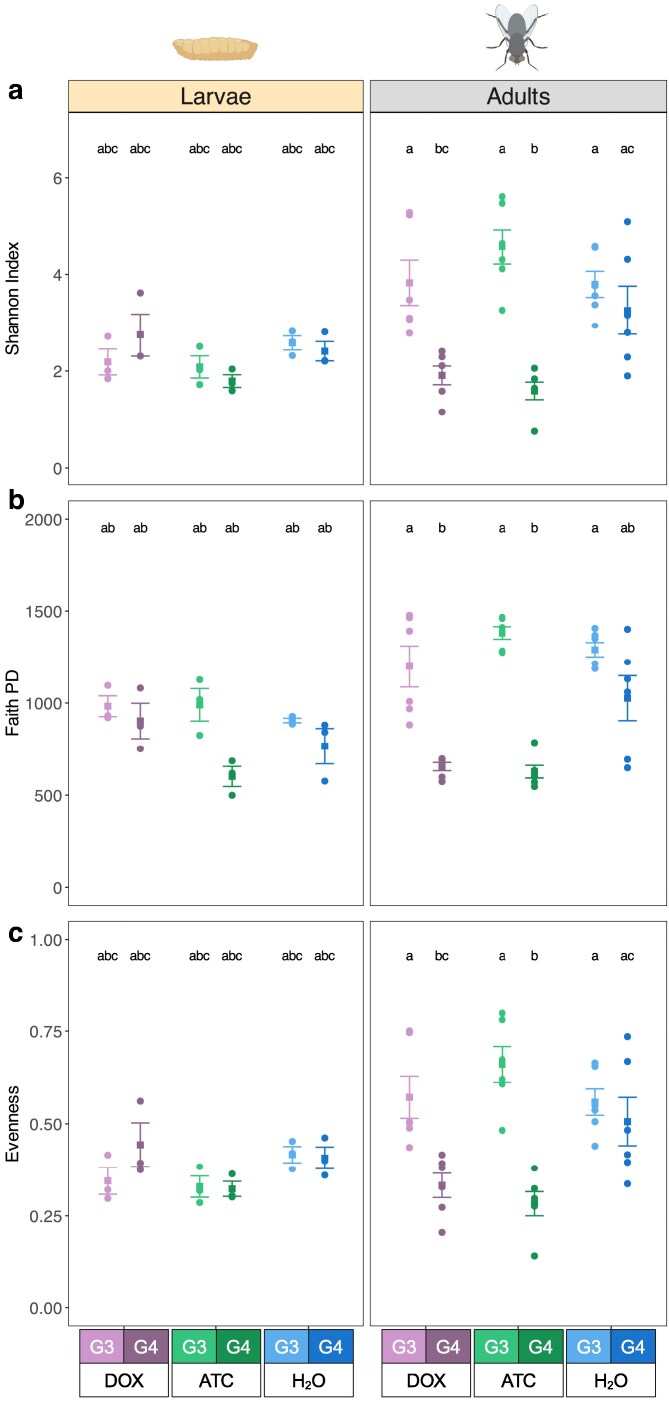
Alpha diversity comparisons. a) Shannon Index. b) Faith PD. c) Pielou’s Evenness. Samples are grouped according to developmental stage, G1-G3 diet, and generation (G3 vs G4). Circles indicate individual data points; squares indicate the mean for each comparison; error bars indicate +/− standard error. Statistically significant differences in group means are denoted by letter combinations (*P* < 0.05, pairwise Wilcoxon rank-sum test with Benjamini–Hochberg correction for multiple comparisons).

We next measured alpha and beta diversity to gain a deeper understanding of the direct and transgenerational impacts of DOX and ATC on the microbiome. These diversity measures take into account the proportional representation of taxa in a sample, not just whether a given taxon is present or absent. Alpha (within-sample) diversity was computed using 3 metrics: the Shannon Index, Faith's phylogenetic diversity (PD), and Pielou's evenness. Pairwise comparisons of adult and larval samples revealed no differences in any alpha diversity metric among G3 flies reared on different diets (Fig. 4a-c). However, the G4 DOX and G4 ATC adults had significantly lower alpha diversity than G3 DOX and G3 ATC adults, respectively, for each of the 3 metrics (*P* < 0.03 for all comparisons) (Fig. 4a-c). We did not observe statistically significant changes in any alpha diversity metric when comparing the G3 and G4 control (H_2_O) samples. These findings suggest that removal of ATC and DOX from the diet of antibiotic-acclimated lines leads to reduced alpha diversity in the next generation of adult flies. We next analyzed beta (between-sample) diversity to assess how similar the experimental groups' microbiomes were to each other. Pairwise comparisons of beta diversity using PERMANOVA reported no significant differences among larval samples, regardless of generation, diet, or stage (Supplementary Table 1). We also observed no differences in beta diversity among adults due to sex (Supplementary File 2). The G4 DOX adults differed significantly from all other adult groups in terms of beta diversity, including the parental G3 DOX adult group (*P* = 0.033) (Supplementary Table 1). Similarly, G4 ATC adults differed significantly from all other adult groups, with the exception of the G4 H_2_O adults, where the difference in beta diversity was not statistically significant (*P* = 0.076). Principal coordinates analysis (PCoA) of the microbiome samples revealed that G4 DOX and G4 ATC adults clustered separately from all other groups in the dataset (Supplementary Fig. 1). Taken together, these findings indicate that removing tetracyclines from acclimated *L. cuprina* lines causes the next generation of adults to possess compositionally distinct, but less diverse, microbiomes.

**Fig. 5.**
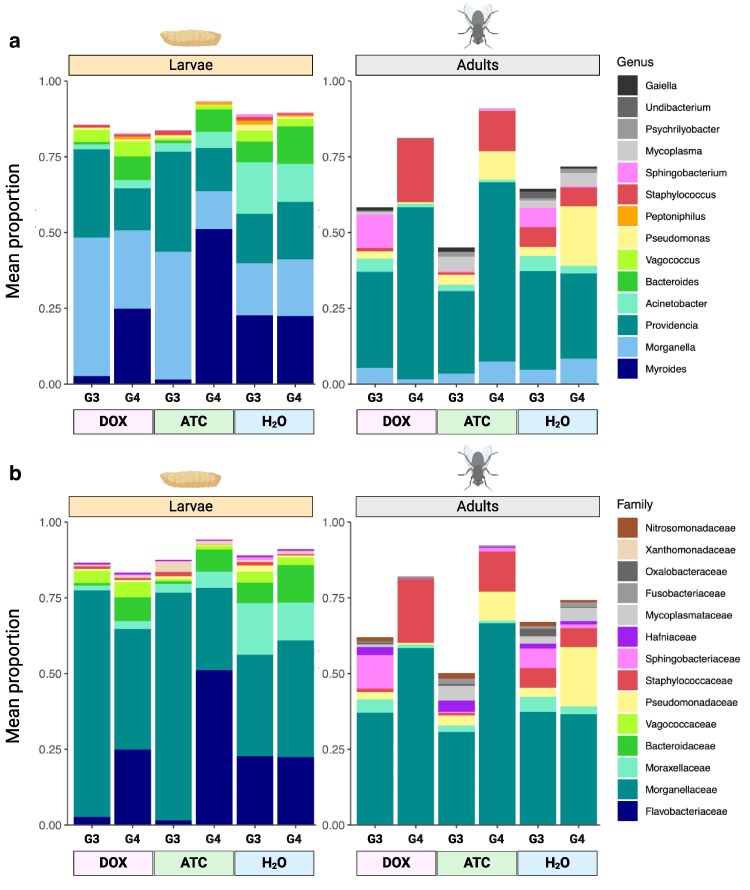
Relative abundance of dominant bacterial taxa in DOX- and ATC-reared flies and their offspring. a) Dominant bacterial genera. Colored bars display the mean proportions of the 10 most abundant genera in larval and adult samples. White space above the bars represents all other taxa (not shown). b) Dominant bacterial families.

We next asked which taxa were driving the observed differences in microbiome composition in the DOX- and ATC-treated G3 flies and their untreated G4 offspring. First, to visualize potential shifts among the dominant bacterial taxa in *L. cuprina*, we generated bar plots displaying the mean proportions of the 10 most abundant genera and families in larval and adult samples (Fig. 5). DOX and ATC appeared to exert broadly similar effects on dominant taxa at both the genus and family level (Fig. 5a, Fig. 5b). Among larvae, G3 DOX and G3 ATC samples closely resembled each other, with proportionally less *Myroides* and *Bacteroides*, and more *Providencia* and *Morganella*, than H_2_O-reared controls (Fig. 5a). Among adults, the G4 DOX and G4 ATC samples also appeared similar, with higher proportions of *Providencia* and *Staphylococcus* and depletion of several less-abundant genera relative to controls (Fig. 5a).

We next conducted a quantitative analysis of differentially abundant taxa that incorporated all 1,933 taxa identified in this study. Pairwise comparisons were performed with ANCOM-BC2 to detect differentially abundant taxa in G3 and G4 DOX and ATC samples relative to H_2_O controls. In addition, ANCOM-BC2 was used to detect missing taxa (structural zeroes). Adult males and females were analyzed separately in order to identify taxa that might be associated with the loss of sexual competitiveness we observed in G3 and G4 DOX males. The results of this analysis are summarized in Supplementary Table 2. Among larvae, DOX and ATC treatment led to significant reductions in the relative abundance of *Myroides* and *Sphingobacterium* in G3 samples (Table 1). DOX treatment was also associated with lower *Acinetobacter* abundance in G3 and G4 larval samples (Table 1). Among adults, no differentially abundant taxa were identified in DOX or ATC samples in G3 (Table 2). However, in G4, DOX adult males and females had 7 and 13 differentially abundant taxa, respectively, relative to H_2_O-treated controls. Among G4 ATC adult samples, we identified only one differentially abundant taxon, *Bacillus*, in the females, which was increased in G4 DOX adult females as well. The relative abundances of *Myroides*, *Peptoniphilus,* and an unresolved ASV (Class: AKAU4049) were decreased in both male and female G4 DOX adults (Table 2). Males, but not females, also displayed decreases in *Serratia*, *Sphingobacterium*, *Acinetobacter*, and *Streptococcus*. Several of these taxa were among the top 20 most abundant taxa in the control groups and may therefore be important constituents of the normal microbiome in *L. cuprina*. For example, the relative abundance of *Myroides*, the single most abundant taxon in control larvae and the 13th most abundant taxon in control adults, was decreased by 94 and 89% among G3 DOX and G3 ATC larvae, respectively (Table 1), and by approximately 99% in G4 DOX adults of both sexes (Table 2). Similarly, the relative abundance of *Sphingobacterium*, the eleventh most abundant taxon in larvae and sixth most abundant taxon in adults, was reduced by 86%, 91%, and 96% in G3 ATC larvae, G3 DOX larvae, and G4 DOX adult males, respectively.

**Table 1.** Differentially abundant taxa in larvae.

Comparison	Family	Genus	Taxon abundance rank	Log fold change ± SE	q-Value
G3 ATC larvae vs G3 H2O larvae	Flavobacteriaceae	Myroides	1	−2.79 ± 0.26	7.15E-06
	Hafniaceae		10	1.70 ± 0.22	1.74E-04
	Sphingobacteriaceae	Sphingobacterium	11	−2.07 ± 0.17	1.41E-06
	Peptostreptococcaceae	Peptostreptococcus	13	−1.38 ± 0.31	2.65E-02
	Enterobacteriaceae		30	1.84 ± 0.23	1.76E-04
	Yersiniaceae		37	1.83 ± 0.30	1.77E-03
G3 DOX larvae vs G3 H2O larvae	Flavobacteriaceae	Myroides	1	−2.23 ± 0.25	4.75E-05
	Sphingobacteriaceae	Sphingobacterium	11	−2.40 ± 0.21	3.45E-06
	Erysipelotrichaceae	Erysipelothrix	15	−1.92 ± 0.43	2.68E-02
	Moraxellaceae	Acinetobacter^1^	18	−4.16 ± 0.33	1.64E-04
	Rikenellaceae	DMER64	206	2.50 ± 0.45	2.11E-02
G4 DOX larvae vs G4 H2O larvae	Moraxellaceae	Acinetobacter^2^	4	−1.91 ± 0.35	4.99E-03

Superscripts (^1,^  ^2,^ etc.) denote different ASVs. Log fold change ± SE = log fold change (natural log) plus or minus the standard error. *q*-Values = *P*-values from ANCOM-BC2 pairwise directional test adjusted for multiple comparisons using the Holm–Bonferroni method.

**Table 2.** Differentially abundant taxa in adults.

Comparison	Family	Genus	Taxon abundance rank		Log fold change ± SE		q-value	
			M	F	M	F	M	F
G4 DOX adults vs G4 H2O adults	Sphingobacteriaceae	Sphingobacterium	6	5	−3.24 ± 0.78	ns	2.84E-02	ns
	Flavobacteriaceae	Myroides	9	15	−5.00 ± 0.61	−4.40 ± 0.69	2.17E-04	1.00E-03
	Yersiniaceae	Serratia	19	24	−3.19 ± 0.79	ns	3.64E-02	ns
	Gemmatimonadaceae	Gemmatimonas	27	34	ns	−3.40 ± 0.53	ns	2.12E-03
	Comamonadaceae		35	41	ns	−2.76 ± 0.49	ns	4.13E-03
	Streptococcaceae	Streptococcus^1^	41	49	−2.75 ± 0.59	ns	1.65E-02	ns
	Peptoniphilaceae	Peptoniphilus	54	60	−2.73 ± 0.57	−2.74 ± 0.58	1.08E-02	1.21E-02
	Longimicrobiaceae	YC-ZSS-LKJ147	59	74	ns	−2.82 ± 0.53	ns	1.35E-02
	Moraxellaceae	Acinetobacter	75	78	−3.23 ± 0.61	ns	1.07E-02	ns
	Class: AKAU4049		77	87	−4.48 ± 0.63	−2.42 ± 0.53	8.18E-03	4.55E-02
	Order: Lactobacillales		137	195	ns	2.23 ± 0.53	ns	2.25E-02
	Pasteurellaceae		149	130	ns	2.32 ± 0.56	ns	2.46E-02
	Streptococcaceae	Streptococcus^2^	187	233	ns	2.53 ± 0.42	ns	1.23E-03
	Streptococcaceae	Streptococcus^3^	331	304	ns	2.87 ± 0.55	ns	2.38E-02
	Veillonellaceae	Veillonella	442	330	ns	3.44 ± 0.60	ns	2.39E-02
	Bacillaceae	Bacillus	575	543	ns	2.87 ± 0.57	ns	1.81E-02
	Campylobacteraceae	Campylobacter	1042	912	ns	2.83 ± 0.56	ns	1.93E-02
G4 ATC adults vs G4 H2O adults	Bacillaceae	Bacillus	575	543	ns	2.90 ± 0.57	ns	1.81E-02

Superscripts (^1,^  ^2,^ etc.) denote different ASVs. Log fold change ± SE = log fold change (natural log) plus or minus the standard error. *q*-Values = *P*-values from ANCOM-BC2 pairwise directional test adjusted for multiple comparisons using the Holm–Bonferroni method.

Detection of structural zeroes (i.e. missing taxa) using ANCOMBC-2 revealed severe losses of microbial diversity in G4 DOX and G4 ATC adults (Supplementary Table 2); 1,044 and 766 taxa were missing from G4 DOX adult male and female samples, respectively, but present in the corresponding H_2_O control groups. The G3 DOX adult male and female samples, in contrast, had 150 (male) and 263 (female) missing taxa relative to controls. A similar but less extreme pattern of transgenerational loss of taxa was seen in the ATC samples, where 866 and 537 missing taxa were identified in G4 ATC adult males and females, respectively, as opposed to 155 and 143 missing taxa in G3 ATC adult males and females (Supplementary Table 2). Given that there were 1,933 total taxa identified in this study, the missing taxa in the G4 DOX and G4 ATC adult samples represent a major loss of bacterial diversity. Additionally, the G4 DOX adults, unlike the G4 ATC adults, were missing several common taxa, including *Bacteroides*, *Vagococcus*, and three ASVs in the genus *Mycoplasma*, each of which was among the top 25 most abundant taxa in adult controls (Supplementary File 2). The results of our quantitative analysis show that DOX and ATC exert both direct and transgenerational effects on microbiome composition in *L. cuprina*, with DOX in particular leading to marked depletion of common and rare taxa in G4.

### Doxycycline and anhydrotetracycline disrupt gene expression in treated flies and untreated offspring

To investigate how DOX and ATC affected gene expression in *L. cuprina* directly and transgenerationally, we sequenced mRNA from G3 and G4 larvae and adults. Read mapping resulted in a total of 15,717 transcripts across 72 samples, 13,927 of which mapped to protein-coding genes. Additional information regarding read mapping and transcript categories can be found in Supplementary File 3. Principal components analysis of the transcriptome data revealed that the samples clustered primarily by stage and secondarily by sex (Supplementary Fig. 2).

**Fig. 6.**
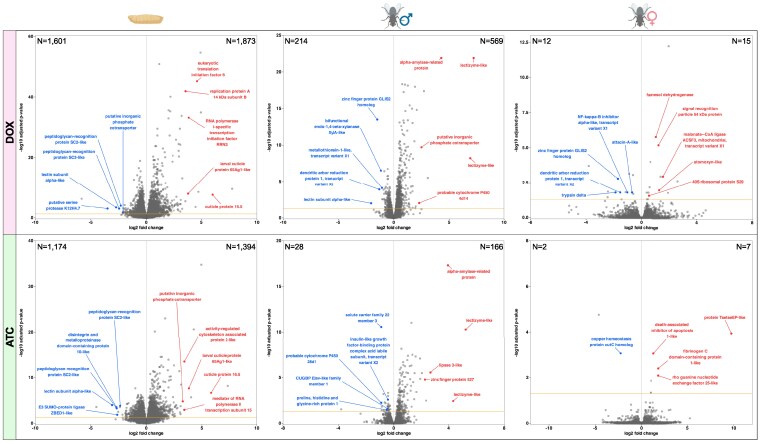
Differential gene expression in third-generation (G3) *L. cuprina* reared on DOX or ATC. Each volcano plot shows the adjusted log2 fold change (*x*-axis) plotted against the adjusted *P*-value (*y-*axis) for genes in the indicated group (larvae, adult males, or adult females) compared to the control diet. For each comparison, the numbers of downregulated and upregulated genes are displayed in the upper-left and upper-right corners of the plot, respectively. The 5 most upregulated and 5 most downregulated genes (based on the absolute value of the log2 fold change) with informative gene IDs are labeled in red (right side of panel) and blue (left side of panel), respectively. The horizontal line above the *x*-axis denotes an adjusted *P*-value cutoff of 0.05. Genes falling below this cutoff were not considered statistically significant and were not labeled.

**Fig. 7.**
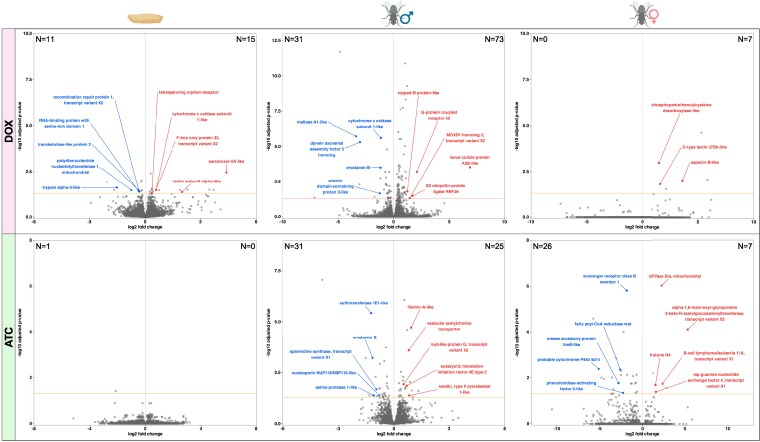
Differential gene expression in the fourth-generation (G4) offspring of *L. cuprina* reared for 3 generations on DOX or ATC. Each volcano plot shows the adjusted log2 fold change (*x*-axis) plotted against the adjusted *P*-value (*y*-axis) for genes in the indicated group (larvae, adult males, or adult females) compared to the control diet. For each comparison, the numbers of downregulated and upregulated genes are displayed in the upper-left and upper-right corners of the plot, respectively. The 5 most upregulated and 5 most downregulated genes (based on the absolute value of the log2 fold change) with informative gene IDs are labeled in red (right side of panel) and blue (left side of panel), respectively. The horizontal line above the *x*-axis denotes an adjusted *P*-value cutoff of 0.05. Genes falling below this cutoff were not considered statistically significant and were not labeled.

DESeq2 was used to identify differentially expressed genes (DEGs) in DOX and ATC samples by comparing them to control (H_2_O) samples from the same generation, stage, and (for adults) sex. The DEGs associated with these 12 comparisons are listed in Supplementary File 3. The log2 fold changes and *P*-values associated with genes from each comparison are visualized in volcano plots (Figs. 6 and 7). We identified many more DEGs in G3 (Fig. 6) than in G4 (Fig. 7), presumably because G3 flies were directly exposed to DOX and ATC, while G4 flies were exposed indirectly through their parents' diets. G3 larvae had the strongest shifts in gene expression of any samples in our dataset, with both DOX and ATC larvae in G3 displaying thousands of differentially expressed genes relative to H_2_O controls (Fig. 6). In contrast, G4 DOX and ATC larvae had >99% fewer DEGs than their G3 predecessors (Fig. 7). Among adults, males always displayed more DEGs than females, possibly because larger inter-sample variability among females reduced our power to detect statistically significant differences (Supplementary Fig. 2). Regardless of stage, sex, or generation, DOX samples generally had more DEGs than ATC samples (Figs. 6 and 7), suggesting that DOX disrupted gene expression more than ATC, despite being used at a lower dose in our study.

To gain a more detailed understanding of the physiological consequences of DOX and ATC treatment in *L. cuprina*, we performed gene ontology (GO) term enrichment analysis on DEGs from each comparison. Lists of enriched GO terms for each comparison are provided in Supplementary File 3. Among G3 larvae, the upregulated genes in both DOX and ATC samples were highly enriched for GO terms associated with protein synthesis and processing (e.g. “translation,” “ribosome,” “protein maturation,” “peptide biosynthetic process,” etc.) (Supplementary File 3). Tetracyclines are known to inhibit protein synthesis in bacteria and mitochondria (Rasmussen et al. 1991; Chopra and Roberts 2001), and DOX has been shown to disrupt the balance of mtDNA and nuclear DNA-encoded mitochondrial gene expression in several eukaryotes (Moullan et al. 2015). Therefore, the upregulation of protein synthesis–related genes we observed in G3 DOX and ATC larvae may represent the cell's attempt to compensate for mitochondrial translation deficits by producing more proteins involved in respiration and other mitochondrial functions. Consistent with this explanation, we found dozens of upregulated, nuclear DNA-encoded genes with mitochondrial functions and localizations among the G3 DOX and ATC larvae. These included several ATP synthase subunit genes, several NADH dehydrogenase genes, and over a dozen mitochondrial 28S ribosomal subunit genes (Supplementary File 3). We also observed that several tRNA- and rRNA-related GO terms were enriched among the upregulated genes in G3 DOX larvae but not G3 ATC larvae, including “rRNA processing,” “maturation of SSU-rRNA,” “tRNA processing,” “tRNA aminoacylation,” “tRNA binding,” etc. DOX, unlike ATC, blocks protein synthesis directly by binding to the aminoacyl-tRNA binding site in the ribosome (Chopra and Roberts, 2001), possibly triggering compensatory upregulation of ribosome-associated RNAs. Among the downregulated genes in G3 DOX and G3 ATC larvae, we observed many GO terms relating to phosphorylation (e.g. “kinase activity,” “protein phosphorylation,” “dephosphorylation,” etc.). DOX is known to inhibit protein phosphorylation in mouse and human cell lines (Bernardino et al. 2009; Han et al. 2014); ATC evidently has a similar effect in *L. cuprina*. We also observed that GO terms potentially related to immunity and defense, such as “autophagy” and “cellular response to stimulus”, were enriched among the set of downregulated genes in G3 DOX and ATC larvae (Supplementary File 3). Indeed, we found that dozens of genes implicated in insect immunity were downregulated in these samples, including antimicrobial peptides (diptericins and attacins), lectin-domain containing proteins, trypsins, mucins, *hemocytin*, serine proteases, and peptidoglycan-recognition proteins (Gillespie et al. 1997; Muhlia-Almazán et al. 2008; Terra et al. 2018; Hu et al. 2024). A smaller number of genes with potential roles in immunity were upregulated in both DOX and ATC larvae, including the predicted antimicrobial peptide *acanthoscurrin* (Lorenzini et al. 2003) and several serine proteases and serine protease inhibitors. Taken together, these findings show that DOX and ATC lead to broadly similar shifts in gene expression in directly exposed (G3) *L. cuprina* larvae, disrupting gene pathways involved in immunity, mitochondrial processes, protein synthesis, and phosphorylation.

We were unable to identify enriched GO terms in G4 larvae due to the small number of DEGs in these samples (Supplementary File 3). The sole DEG in the G4 ATC larvae was an uncharacterized transcript with strong sequence homology to the *L. sericata* gene *putative cyclin-dependent serine/threonine-protein kinase*. This transcript was highly downregulated in both ATC and DOX larvae in G4 (>75% reduction in both groups relative to controls). The downregulation of this putative protein kinase is consistent with the downregulation of phosphorylation-related transcripts observed in G3 larvae and suggests that both DOX and ATC influence phosphorylation transgenerationally as well as directly. Unexpectedly, we also found that 2 transcripts predicted to encode proteins with antibacterial activity, *sarcotoxin-2A-like* and *lectin subunit alpha-like*, were strongly upregulated in G4 DOX larvae, contrasting with the widespread downregulation of defense-related genes observed in G3 larvae (Supplementary File 3). Several other genes displayed similarly inverted responses to DOX across generations (downregulated in G3 but upregulated in G4, or vice versa). We identified only one gene that was consistently down- or upregulated in both generations: *trypsin alpha-3-like*, a proteolytic enzyme that is upregulated in response to protist infection in bumblebees (Giacomini et al. 2023). The expression of this gene was reduced by >50% in both G3 and G4 DOX larvae relative to controls. Overall, these results suggest that tetracyclines exert complex transgenerational effects on gene expression in larvae.

We next turned our attention to patterns of gene enrichment in adult samples. The upregulated genes in DOX and ATC adult males in G3 were highly enriched for mitochondria-associated GO terms (“oxidoreductase activity,” “mitochondrion,” “acyl-CoA dehydrogenase activity,” etc.), as well as numerous metabolism- and catabolism-related Biological Process (GO:BP) terms (Supplementary File 3). Many highly upregulated transcripts were shared by DOX and ATC males in G3, including genes with known or predicted roles in insect immunity, including *peptidoglycan-recognition protein SC2* (Bischoff et al. 2006), *hemocytin* (Hu et al. 2024), and several serine protease and lectizyme-like genes (Yassine et al. 2014; Geiger et al. 2015). We also identified several downregulated transcripts in G3 DOX males with probable roles in immunity, including *lectin subunit alpha*, which is upregulated in response to fungal infection in houseflies (Li et al. 2022), and *diptericin-D*, an antibacterial peptide produced by several fly species in response to infection (Imler 2014; McKenna et al. 2022). G3 ATC adult males shared the majority (57%) of their downregulated genes with G3 DOX adult males. These shared downregulated genes had predicted roles in phosphorylation, transcription initiation, and mRNA processing, among other functions. The G3 DOX and ATC female samples had too few DEGs to identify enriched GO terms, but were similar in some respects to their male counterparts. For example, several immunity-related transcripts were differentially expressed in DOX G3 females, including antimicrobial peptides *attacin-A* (Hu and Aksoy 2005) and *stomoxyn* (Boulanger et al. 2002). Similarly, we found that genes involved in mitochondrial fatty acid synthesis, as well as several genes coding for eukaryotic ribosome subunits, were upregulated in both male and female G3 DOX adults (Supplementary File 3). While the G3 ATC females shared only one DEG with G3 ATC males, both sexes displayed a general pattern of upregulation of putative immunity-related genes. The most striking example of this was the >9-fold (>850x) increase in the expression of *Tsetse-EP-like* in G3 ATC females relative to H_2_O-treated female controls (Fig. 6, Supplementary File 3). In the tsetse fly *G. morsitans*, Tsetse EP protects flies from trypanosome establishment in the midgut and is also upregulated in response to *E. coli* infection (Haines et al. 2005; Haines et al. 2010). Taken together, these results suggest that direct exposure to DOX and ATC induces similar transcriptional responses in G3 adults and larvae, namely widespread shifts in immune gene expression and the upregulation of mitochondria-associated genes.

Dozens of DEGs were identified in the G4 DOX and ATC adult samples (Fig. 7, Supplementary File 3), demonstrating transgenerational disruption of gene expression due to tetracyclines. Despite this, relatively few of the DEGs observed in G3 (*N* = 13) were also differentially expressed in G4. We found that the nuclear DNA-encoded mitochondrial genes *cytochrome c oxidase subunit NDUFA4* and *dihydrolipoyl dehydrogenase* were upregulated in DOX and ATC males, respectively, in both G3 and G4, suggesting persistent disruption of mitochondrial function following antibiotic treatment. In DOX adults, as in DOX larvae, we found that some genes which were downregulated in G3 were upregulated in G4, including the microtubule-associated protein *futsch*, whose homolog in *D. melanogaster* plays important roles in the maintenance of adult neurons (da Cruz et al. 2005). The G4 DOX males and G4 ATC males shared 7 downregulated and 11 upregulated genes, among which were genes with predicted roles in proteolysis, phosphorylation, actin binding, and transcriptional regulation. Both groups also downregulated the expression of a nuclear DNA-encoded pseudogene for the mitochondrial cytochrome c oxidase I subunit, and upregulated the expression of the mitochondrial cation exchange protein *Letm1* (Jiang et al. 2009).

The G4 DOX and G4 ATC females shared no DEGs with each other, nor with their G3 female parents (Supplementary File 3). A small number of DEGs were shared between G4 DOX males and females, and between G4 ATC males and females (*N* = 1 and *N* = 2 genes, respectively), but these were uncharacterized genes with unknown functions. Among the handful of annotated DEGs in the G4 DOX female samples were 2 upregulated genes with probable roles in defense against pathogens, *sapecin-6* and *C-type lectin 37Db-like*. Sapecins encode antimicrobial peptides and are upregulated in response to immune stimulation in the flesh fly *Sarcophaga bullata* (Mášová et al. 2009), while C-type lectins promote the defensive melanization response in *Drosophila* (Ao et al. 2007). Among the G4 ATC female adults, we observed differential expression of several genes likely to be involved in carbohydrate synthesis, detoxification, and the defensive melanization response. For example, the melanization-related genes *mgl* (Riedel et al. 2011) and *phenoloxidase-activating factor 2-like* (Nakhleh et al. 2017) were downregulated in G4 ATC females relative to H_2_O controls. We also observed dysregulation of multiple mitochondrial genes, including the upregulation of *GTPase Era,* which is essential for mitochondrial ribosome assembly (Gruffaz and Smirnov 2023), and downregulation of *carnitine O-palmitoyltransferase* (*whd*), which is required for proper fatty acid metabolism in *Drosophila* (Strub et al. 2008). These results indicate that, as in adult males, DOX and ATC transgenerationally perturb the expression of mitochondrial and immunity-related genes in adult female blowflies.

### Gene expression patterns associated with reduced sexual competitiveness in DOX-treated adult males and their offspring

Given the reduced sexual competitiveness in DOX-treated G3 adult males and their untreated G4 sons described above, we wished to examine the transcriptional profiles of these males in greater detail. To identify genes associated with decreased sexual competitiveness, we analyzed the subset of DEGs found in G3 and/or G4 DOX adult males but not in G4 or G3 ATC adult males. This revealed 614 and 84 DEGs exclusive to G3 and G4 DOX adult males, respectively (Supplementary File 3). Of the three DEGs that were consistently down- or upregulated in both generations, only one was characterized: *cytochrome c oxidase subunit NDUFA4*, an essential component of the electron transport chain in mitochondria (Balsa et al. 2012), which was upregulated by approximately 25% in both G3 and G4 DOX adult males. In G3 DOX males, the most differentially expressed genes (based on the magnitude of log2 fold change) included multiple genes with putative roles in immunity and detoxification, including *cytochrome P450 4d14*, two *arylphorin subunit C223-like* transcripts, *trypsin-like* and *carboxypeptidase B*, *bifunctional endo-1,4-beta-xylanase XylA-like* and *lectin subunit alpha-like*. Similarly, several genes with potential immunity-related roles were differentially expressed in the G4 DOX adult males, such as *larval cuticle protein A2B*, which is upregulated in bees in response to viral infection (Deng et al. 2020) and which was steeply upregulated (log_2_fc = 6.85) in our G4 DOX males relative to H_2_O-treated controls. In addition, multiple genes with known or potential roles in male sexual behavior were differentially expressed in the G3 and G4 DOX adult male samples. These included *G-protein coupled receptor 52*, a homolog of the *DopEcr* gene essential for sex pheromone responses in male moths (Abrieux et al. 2013); *choline O-acetyltransferase*, which regulates male courtship behavior in *Drosophila* (Huang et al. 2016); *tipE*, which is necessary for normal courtship song production in *Drosophila* (Peixoto and Hall 1998) and implicated in male mating success in the wasp *Nasonia* (Diao et al. 2016); and *protein ITPRID2* (*olf186-M*), which is upregulated in male *Drosophila* during courtship (Ellis and Carney 2011). These findings suggest that the reduction in sexual competitiveness in DOX-treated males and their offspring may have been due to the disruption of gene pathways involved in courtship, immunity, and/or cellular respiration.

## Discussion

Tetracyclines are important and widely used antibiotics, but they are associated with a variety of negative side effects in diverse eukaryotes. Emerging evidence shows that tetracyclines can cause side effects that persist even after tetracyclines are removed, including transgenerational effects that appear in the untreated offspring of treated individuals. In this study, we found that 2 tetracyclines, doxycycline (DOX) and anhydrotetracycline (ATC), disrupted gene expression and microbiome composition directly and transgenerationally in the blowfly *L. cuprina.* In addition, we found that doxycycline reduced male sexual competitiveness directly and transgenerationally, which has important implications for genetic pest management.

In many insect species, the microbiome contains essential symbiotic bacteria. While direct exposure to tetracyclines is known to reduce or eliminate symbionts (Dedeine et al. 2001; Boucias et al. 2012; Weiss et al. 2013; Raymann et al. 2017), much less is known about the transgenerational impacts of tetracyclines on the insect microbiome. As both DOX and ATC are broad-spectrum antibiotics, we expected that flies treated with these compounds would have fewer and less diverse bacteria in G3 and that the microbial phenotype would recover to some degree in G4 after antibiotics were removed. While DOX and ATC did indeed alter microbiome composition in G3 larvae and adults, the most severe microbiome disruption was, unexpectedly, observed in G4 adults. Both DOX and ATC adults in G4 had less diverse microbiomes than their G3 parents, higher bacterial DNA levels (as measured by qPCR), and distinct microbiome composition relative to all other groups. G4 DOX adults, in particular, displayed severe shifts in microbiome composition, with many common taxa missing or highly reduced in these samples. Our results are inconsistent with a simple model of antibiotic activity in which the microbiome is fully or partially restored after tetracyclines are removed. We instead speculate that the dysbiosis we observed in the G4 adults was a consequence of tetracyclines perturbing the native microbiome in G1-G3, which created opportunities for some resident taxa to overproliferate and displace other taxa after antibiotics were removed in G4. We found that facultative anaerobes like *Providencia* and *Staphylococcus* were overrepresented in G4 DOX and ATC adults compared to controls. Overproliferation of facultative anaerobes has been observed in humans and mice after antibiotic cessation, along with reduced bacterial diversity (Pham and Lawley 2014). Likewise, antibiotic treatment and subsequent removal have been shown to cause dysbiosis in insects, leading to increases in the relative abundance of native bacteria (Raymann et al. 2017; Li et al. 2020). Our results demonstrate that antibiotics can also induce dysbiosis transgenerationally in *L. cuprina*, with DOX appearing to be a particularly potent disruptor of the microbiome.

In addition to targeting bacteria, tetracyclines target eukaryotic mitochondria due to their shared eubacterial ancestry (Clark-Walker and Linnane 1966; Moullan et al. 2015). A common mitochondrial response to stress is genome replication (i.e. increased mtDNA copy number) (Malik and Czajka 2013; D’Erchia et al. 2015). Although antibiotics, including tetracyclines, are known to induce mitochondrial stress in a variety of organisms (Moullan et al. 2015; Luger et al. 2018; Xiao et al. 2019), their impact on mtDNA is less clear, with studies variously reporting decreases (Hangas et al. 2018) or no change (Hsiao et al. 2010) in mtDNA levels as a consequence of antibiotic treatment. While less research has been conducted on the transgenerational effects of antibiotics on mtDNA, steeply elevated mtDNA levels were observed in the fly *Drosophila simulans* 2 generations after tetracycline exposure (Ballard and Melvin 2007). To better understand the effect of tetracyclines on mitochondrial DNA replication, we used qPCR to measure mtDNA in G3 DOX- and ATC-treated flies and their untreated G4 offspring. We found that neither antibiotic affected mtDNA copy number in either generation. The aforementioned *D. simulans* study found that *Wolbachia* infection shielded flies from tetracycline-induced changes in mtDNA copy number. However, although *Wolbachia* is a common endosymbiont in many Diptera (de Oliveira et al. 2015), it was not present in any of our 16S DNA samples, which is consistent with previous reports that failed to find *Wolbachia* in *L. cuprina* (Mingchay et al. 2014; Deel et al. 2022).

Although DOX and ATC did not affect mtDNA copy number, these compounds clearly influenced mitochondria at the organismal level, as our transcriptome analysis revealed widespread upregulation of nuclear DNA-encoded mitochondrial genes in response to tetracyclines. This upregulation of mitochondrial genes was most pronounced in G3 larvae, followed by G3 adults, which was expected given that the G3 flies were directly exposed to tetracyclines through their diet. The higher number of differentially expressed genes observed in G3 larvae relative to G3 adults may be a consequence of the larvae ingesting more antibiotics than adults, as larvae consume several times their body weight in meat. A number of mitochondrial genes were also upregulated in G4 adults, including genes essential for mitochondrial ribosome assembly (*GTPase Era*) (Gruffaz and Smirnov 2023), the electron transport chain (*cytochrome C oxidase subunit NDUFA4*) (Balsa et al. 2012), and regulation of ATP synthesis (*letm1*) (Jiang et al. 2009). Although some studies have found that nuclear DNA-encoded mitochondrial genes are generally downregulated in response to DOX (Ballard and Melvin 2007), our findings are broadly consistent with results showing mitochondrial gene upregulation in response to DOX (Rao et al. 2012; Lamb et al. 2015; Dijk et al. 2020) and ATC (Mottis et al. 2022). This upregulation of nuclear DNA-encoded mitochondrial genes may be the cell's attempt to compensate for impaired mitochondrial translation, which is a well-known side effect of tetracyclines (Moreno-Lastres et al. 2012; Moullan et al. 2015). G4 flies upregulating mitochondrial genes could indicate that these untreated offspring are still experiencing mitochondrial stress, which could have negative implications for genetic biocontrol programs, as mitochondrial function is an important component of an organism's overall fitness (Chatzispyrou et al. 2015).

In addition to disrupting mitochondrial gene expression, DOX and ATC directly and transgenerationally perturbed the expression of numerous genes with known or predicted roles in defense and immunity in insects. Somewhat surprisingly, we found that several genes involved in pathogen defense were among the most upregulated genes in our entire dataset. These included several predicted *lectizyme* transcripts in G3 DOX and ATC adult males, *Tsetse EP* in G3 ATC adult females, *sarcotoxin-2A* in G4 DOX larvae, and *sapecin-B* in G4 DOX adult females. The first 2 genes are known to be upregulated in response to infection with trypanosomes (*lectizyme* and *tsetse EP*) or bacteria (*tsetse EP*) in tsetse flies. *Sarcotoxin-2A* and *sapecin-B* are antibacterial proteins upregulated in response to infection or physical injury in a variety of insects, including blowflies (Ando and Natori 1988; Mášová et al. 2009; McKenna et al. 2022). The strong upregulation of these genes in our dataset is notable because antibiotics often reduce the expression of antimicrobial effector genes (Rao et al. 2012; Galarza et al. 2021), presumably because the antibiotics kill pathogenic bacteria before they can elicit an immune response. Indeed, we observed strong downregulation of antimicrobial peptides and other immunity-related genes in our G3 DOX and ATC samples. We speculate that the mixed upregulation and downregulation of immunity-related genes observed in this study was due to antibiotic-mediated dysbiosis. Endosymbiotic bacteria are known to play important roles in insect immunity, and removing these bacteria can promote infection with opportunistic pathogens (Oliver and Martinez 2014; Douglas 2015). In honeybees, upregulation of immunity-related genes has been observed in untreated bees receiving dysbiotic microbiomes from tetracycline-treated nestmates (Kowallik et al. 2022). Destabilization of the natural bacterial community in *L. cuprina* due to DOX or ATC treatment may have rendered the flies more susceptible to infection, leading to subsequent upregulation of immune effectors. This effect may have been especially pronounced in G4 because antibiotics were removed and could no longer confer broad protection against bacteria. For example, *Myroides*, which was one of the most abundant bacterial genera in control samples, was nearly eliminated from G4 DOX adults of both sexes. *Myroides* is a constituent of the microbiomes of many Dipteran species (Chavshin et al. 2012; Singh et al. 2015; Hadapad et al. 2019; Zhineng et al. 2021) with antibacterial (Islam et al. 2014) and anti-inflammatory (Dharne et al. 2008) properties, suggesting that it may protect its hosts from outside pathogens. In our study, the antimicrobial genes *sapecin-B* and *C-type lectin 37Db* were strongly upregulated in G4 DOX adult females, while *larval cuticle protein a2b*, which is upregulated in response to viral infection in bees (Deng et al. 2020), was likewise strongly upregulated in G4 DOX adult males. While additional research would be required to confirm whether native bacteria like *Myroides* regulate immune gene expression in *L. cuprina*, our results suggest that the microbiome has immunomodulatory roles in this species, as in other insects.

In this study, we tested 2 antibiotics, doxycycline (DOX) and anhydrotetracycline (ATC). Despite some commercial vendors’ claims that ATC has no antibiotic activity (Takara Bio Inc. 2024), ATC's antibacterial properties have been known for decades (Oliva et al. 1992). However, while numerous studies have examined DOX's effects on microbiome composition, gene expression, and reproduction in diverse organisms (Rao et al. 2012; Darby et al. 2014; Chatzispyrou et al. 2015), ATC's effects are virtually unknown. We found that ATC and DOX exerted broadly similar effects on the microbiome in *L. cuprina*, as treatment with both compounds led to reduced alpha diversity and bacterial DNA levels in G3 relative to G4 adults, as well as similar shifts in the proportions of dominant taxa. In addition, both antibiotics affected many of the same biological pathways, as indicated by shared GO terms, and shifted the expression of many of the same genes. However, only DOX was associated with reduced male sexual competitiveness, despite being used at only a quarter of the dose of ATC. The reduced sexual competitiveness we observed in DOX males may be attributable to DOX's different mode of action. Studies directly comparing the activities of different tetracyclines on various strains and species of bacteria have shown differences in bacterial susceptibility to so-called typical tetracyclines (e.g. DOX) and atypical tetracyclines (e.g. ATC) (Oliva and Chopra 1992; Mottis et al. 2022). Accordingly, we found different sets of differentially abundant and missing taxa in the DOX and ATC microbiome samples. *Myroides*, one of the most common taxa in the control samples, was nearly missing from G4 DOX adults but found at comparable levels to controls in G4 ATC adults. Similarly, the G4 DOX (but not ATC) adult males had lower relative abundances of *Serratia* and *Sphingobacterium* relative to controls. These bacteria have known or suspected roles in anti-pathogen defense and stress tolerance in other insect species (Ganley et al. 2018; Bai et al. 2019; Zhou et al. 2019; Omoke et al. 2021). DOX and ATC males also showed differences at the transcriptomic level, with G3 and G4 DOX males differentially expressing dozens of unique genes, several of which are implicated in male sexual behavior, immunity, and mitochondrial function. Whether the microbiome- and transcriptome-level differences we observed between DOX and ATC males directly influence sexual competitiveness and other fitness parameters remains to be determined.

Our study was designed to mimic the rearing conditions found in biocontrol programs that use Tet-OFF genetic sexing strains to mass-produce males. Accordingly, we reared our flies for multiple generations (G1-G3) on tetracyclines and removed these antibiotics only in the final generation (G4), simulating the removal of tetracyclines used to produce a male-only cohort for a field release. We found that the untreated adult male offspring of DOX-reared flies were approximately 20% less sexually competitive than controls. Models predict that even modest decreases in male sexual competitiveness can reduce the effectiveness of population suppression in Sterile Insect Technique (SIT), female-specific Release of Insects carrying a Dominant Lethal (fsRIDL), and related biocontrol systems (Tsubaki and Bunroongsook 1990; Vreysen et al. 2006; Gentile et al. 2015; Vella et al. 2021). Lower male sexual competitiveness translates into higher operating costs for the biocontrol program, as more males must be reared and released to compensate for lost matings (Mayer et al. 1998).

Importantly, while our study only examined the effects of DOX and ATC on wild-type flies, transgenic insects are often less fit than wild types (Catteruccia et al. 2003; Irvin et al. 2004). The combined effects of tetracyclines and transgenes may render males very unfit compared to wild males in the field. A recent study evaluating the sexual performance of screwworm males from a transgenic female-killing strain (Arp et al. 2024) may serve as an example of this phenomenon. As in our study, screwworms were reared for 3 generations on 25 ug/mL DOX, and male sexual competitiveness was tested in the untreated G4 offspring. The transgenic G4 DOX screwworm males obtained half as many matings with wild-type females as our non-transgenic G4 DOX blowfly males. In addition to potential fitness costs associated with tetracyclines and transgenes, mass-reared males often have to contend with crowding, chilling, travel, radiation (in SIT programs), inbreeding, and other factors that can impair field performance (Mayer et al. 1998; Lux et al. 2002; Iyaloo et al. 2020; Sánchez-Aldana-Sánchez et al. 2023).

Given that many of these factors are necessary for program operation or otherwise difficult to avoid, minimizing the harm from tetracyclines could be important for maintaining adequate male fitness for population suppression. One option might be to switch from using conventional tetracyclines (such as doxycycline or tetracycline) to an atypical tetracycline like ATC, which in our study, did not reduce male sexual competitiveness. However, we note that both ATC and DOX induced similar shifts in microbiome composition and gene expression in *L. cuprina*, warranting further evaluation of ATC's impacts on male health and fitness. Orthologs of some of the most dysregulated genes in our G4 ATC male samples have been shown in other insects to play important roles in neuronal development (Jeong et al. 2017), cuticle formation (Guan et al. 2006), longevity (Hofer et al. 2024), sleep cycle regulation (Bedont et al. 2023), and other biological processes that may impact fitness. In addition, several traits that may be important for male performance in the field were not evaluated in this study, including body size, longevity, and dispersal ability. ATC is also very expensive compared to DOX (priced by one manufacturer at $6,300/g, vs $70/g for DOX, at the time of writing (Thermo Fisher Scientific Inc. 2024)), limiting ATC's practicality as a substitute for DOX in Tet-OFF genetic sexing systems.

Various dietary supplementation strategies have been proposed to mitigate the harm caused by tetracyclines. One option that could be explored in a mass-rearing context is the addition of beneficial bacteria (probiotics) to the diet. In 2 major Dipteran pests, *Bactrocera dorsalis* (Cai et al. 2018) and *Ceratitis capitata* (Gavriel et al. 2011), supplementation with the native bacterium *Klebsiella* reversed fitness declines caused by exposure to radiation. *Proteus mirabilis*, a beneficial bacterium commonly (but not always [Gasz et al. 2021]) found in blowflies and other Calliphorid fly species (Ma et al. 2012; Blenkiron et al. 2015; Singh et al. 2015; Arp et al. 2022), was shown to improve adult emergence and survival when added to the diet of *B. dorsalis* (Khan et al. 2019). Although we did not identify *P. mirabilis* in this study, we noted that other potentially beneficial taxa, such as *Myroides*, *Serratia*, and *Sphingobacterium*, were significantly depleted in G4 DOX male adult samples. These bacteria might be good candidates for dietary supplementation, but additional research would be needed to determine whether they restore male fitness in antibiotic-treated *L. cuprina*. Alternatively, biocontrol programs may be able to avoid antibiotics entirely by developing genetic sexing systems that use different chemicals, such as auxin or quinic acid, to control gene expression (Jaffri et al. 2020). Though not as well-characterized as Tet-OFF systems, auxin-dependent systems can reliably control gene expression in *D. melanogaster* (McClure et al. 2022), and quinic acid-dependent gene expression systems have been successfully developed in *D. melanogaster* (Potter and Luo 2011) and *Caenorhabditis elegans* (Wei et al. 2012). Genetic sexing strains that use temperature, rather than antibiotics, to control gene expression have been developed in the Medfly *C. capitata* (Augustinos et al. 2017), while antibiotic-free, CRISPR-based female-killing and male-sterilizing pgSIT strains have been engineered in *D. suzukii* (Kandul et al. 2022) and the mosquito *Aedes aegypti* (Li et al. 2024). These technologies could be explored as alternatives to Tet-OFF systems for female-killing or transformation in other insect pests, including blowflies and the screwworm.

In conclusion, we identified both direct and transgenerational changes in the microbiome, transcriptome, and male fitness due to treatment with tetracyclines in the blowfly *Lucilia cuprina*. Our findings emphasize the need for researchers to be aware of the effects of long-term antibiotic exposure, which may confound experimental results and jeopardize the success of promising forms of biocontrol. Future work investigating the mechanisms by which tetracyclines alter physiology and behavior could improve our understanding of these important compounds and suggest methods to mitigate their harmful effects.

## Data Availability

The blowfly lines used in this study are available upon request. Supplementary Fig. 1 shows the principal coordinates analysis (PCoA) for the 16S microbiome samples. Supplementary Fig. 2 shows the principal components analysis (PCA) for the transcriptome samples. Supplementary Fig. 3 displays the fly colony rearing and sampling scheme. Supplementary Table 1 contains microbiome beta diversity comparisons. Supplementary Table 2 lists the number of differentially abundant and missing taxa in the microbiomes of different experimental groups. Supplementary Table 3 displays the results of an ATC dosage experiment showing that 100 ug/mL is a sufficient maintenance dose for a fly line carrying a tetracycline-repressible female-lethal gene. Supplementary Table 4 lists all primers used in this study. Supplementary File 1 contains raw data and statistical analyses from the male sexual competitiveness assays. Supplementary File 2 contains data related to the microbiome dataset (16S sample metadata, read counts and taxonomic identifications, and statistical analysis results). Supplementary File 3 contains data related to the transcriptome dataset (metadata, read mapping statistics, raw and normalized read counts, lists of differentially expressed genes, and gene ontology enrichment results). Supplementary File 4 contains all qPCR-related data, including raw data, metadata, calculations, and statistical analyses. Supplemental Material is available at figshare: https://doi.org/10.25387/g3.28528646. The 16S amplicon sequencing reads are available at NCBI Sequence Read Library BioProjectID: PRJNA1118283 (https://www.ncbi.nlm.nih.gov/bioproject/PRJNA1118283/). The blowfly RNAseq reads are available at NCBI Sequence Read Library BioProjectID: PRJNA1227982 (https://www.ncbi.nlm.nih.gov/bioproject/PRJNA1227982). The R code used to analyze and visualize the microbiome and transcriptome data is available at https://github.com/AlexisKriete/tetracyclines-microbiome-transcriptome/. Supplemental material available at G3 online.
